# Dual Inhibition of Ornithine Decarboxylase and A_1_ Adenosine Receptor Efficiently Suppresses Breast Tumor Cells

**DOI:** 10.3389/fonc.2021.636373

**Published:** 2021-03-11

**Authors:** Hongyan Ma, Qizhang Li, Jing Wang, Jing Pan, Zhengding Su, Sen Liu

**Affiliations:** ^1^ National “111” Center for Cellular Regulation and Molecular Pharmaceutics, Key Laboratory of Industrial Fermentation (Ministry of Education), Hubei University of Technology, Wuhan, China; ^2^ Institute of Biomedical and Pharmaceutical Sciences, Hubei Key Laboratory of Industrial Microbiology, Hubei University of Technology, Wuhan, China; ^3^ Hubei Key Laboratory of Tumor Microenvironment and Immunotherapy, Medical College, China Three Gorges University, Yichang, China

**Keywords:** dual inhibitor, ornithine decarboxylase 1 (ODC), A_1_AR, polyamine pathway, cyclic AMP (cAMP) pathway

## Abstract

Personized treatment of breast cancer is still a challenge, and more treatment options for breast cancer are warranted. Combination therapies have been a highly appreciated strategy for breast cancer treatment in recent years, and the development of new combination therapies could improve patient outcomes. Adenosine and polyamines are both endogenous metabolites with indispensable biological functions. Adenosine binds with the A_1_ adenosine receptor (A_1_AR) to downregulate cAMP concentration, and both low cAMP content and high polyamine levels stimulate the growth and proliferation of cancer cells. In this work, we initially used a polyamine synthesis inhibitor, DFMO (*α*-difluoromethylornithine), and an A_1_AR inhibitor, DPCPX (8-cyclopentyl-1,3-dipropylxanthine) to investigate if simultaneously inhibiting A_1_AR and polyamine synthesis has synergistical antitumor effects. Next, we investigated a dual inhibitor (ODC-MPI-2) of A_1_AR and ODC (ornithine decarboxylase 1), the rate-limiting enzyme in polyamine biosynthesis. We investigated if ODC-MPI-2 could inhibit the proliferation and growth of breast cancer cells. Our data showed that DFMO and DPCPX synergistically inhibit the growth and proliferation of MCF-7 cells. We also demonstrated that ODC-MPI-2 reduces cellular polyamine levels and elevates cAMP concentration. We further showed that ODC-MPI-2 inhibits the growth, proliferation, and migration/invasion of MCF-7 cells. Finally, ODC-MPI-2 showed a preference for inhibiting triple-negative breast cancer cells. The dual inhibition of ODC and A_1_AR is a new combination therapy strategy for treating breast cancer, and dual inhibitors of ODC and A_1_AR may be effective future drugs for treating breast cancer.

## Introduction

Cancer is a leading cause of death before 70 years worldwide and breast cancer is second in incidence in males and females (1). Among females, breast cancer is the most commonly diagnosed cancer and the leading cause of cancer death (1). One of the most significant characteristics of breast cancer is its high degree of inter- and intra-tumoral heterogeneity (2). To tackle the complexity of breast cancer, combination therapy has become an important strategy and has been proven to be clinically successful (3). By the combined use of two or more therapeutic agents, combination therapy has many advantages against monotherapy, including enhanced efficacy, lower side effects, and less drug resistance (4). Previous successes of combination therapy also demonstrated that simultaneously targeting multiple signaling pathways is an efficient anti-tumor strategy (5). Therefore, developing more options and alternatives of combination therapy for treating breast cancer is of great value (6).

Adenosine is an endogenous purine nucleoside playing important roles in many physiological processes such as regulating sleep and blood flow, and it has been used as a clinical medication (7). The physiological activities of adenosine are mediated by adenosine receptors (ARs), a kind of G protein-coupled receptors (GPCRs) ubiquitously distributed in almost all cells and important for numerous cellular functions (7). The known AR family has four members, namely A_1_AR, A_2A_AR, A_2B_AR, and A_3_AR. All ARs can be activated by adenosine, but their functions are distinctly diversified. The activation of A_1_R and A_3_R inhibits the adenylyl cyclase activity and reduces the cyclic AMP (cAMP) level, but the activation of A_2A_R and A_2B_R has opposite roles (7). A_1_AR plays beneficial roles in ischemia–reperfusion injury, neurodegenerative diseases, pain, and sleep (8). Meanwhile, A_1_AR antagonists are attracting attention as anti-tumoral agents. For example, Lin et al. (9) demonstrated that the A_1_AR inhibitor DPCPX (8-cyclopentyl-1,3-dipropylxanthine) reduced the proliferation of breast cancer cells; Dastjerdi et al. (10) found that DPCPX induced apoptosis in MCF-7 breast cancer cell line, and Zhou et al. (11) showed that DPCPX inhibited the proliferation and migration of renal cell carcinoma cells.

Polyamines are cationic ligands containing multiple charged nitrogen centers. Spermidine, spermine, and their precursor putrescine are the representative polyamines in mammalian cells. Because polyamines are indispensable for cell growth and proliferation, cellular polyamine levels are strictly modulated by a sophisticated polyamine metabolic network consisting of polyamine synthesis, catabolism, and transport (12). In mammalian cells, polyamine biosynthesis starts from ornithine, a product of arginine in the urea cycle. Ornithine is converted to putrescine by ODC (ornithine decarboxylase 1), the first rate-limiting enzyme for polyamine synthesis. Previous studies found that ODC is often oncogene-driven and a *bona fide* drug target (13, 14). The expression level of ODC and the polyamine concentration are significantly up-regulated in breast cancer cells, and inhibiting ODC is an attracting option for treating breast cancer (15–17). One of the best known ODC inhibitor is DFMO (*α*-difluoromethylornithine), which has been intensively tested in many clinical trials for treating cancers (18). Nonetheless, the clinical use of DFMO is limited by the compensatory uptake of polyamines from the extracellular environment by tumor cells, which prompts the researchers to discover better inhibitors for decreasing cellular polyamine content and inhibiting the growth of cancer cells.

Recently, our group discovered several inhibitors targeting polyamine synthesis (19–21), one of which is a novel multi-purpose ODC inhibitor, ODC-MPI-2 (22). Meanwhile, we noticed that ODC-MPI-2 was previously identified as an A_1_AR inhibitor (23). Considering the importance of adenosine and polyamines for cell viability, we set out to study if ODC-MPI-2 has anti-tumor activity as a dual inhibitor of ODC and A_1_AR in treating breast cancer. We firstly confirmed that the ODC specific inhibitor DFMO and the A_1_AR specific inhibitor DPCPX (8-cyclopentyl-1,3-dipropylxanthine) synergistically inhibit the growth and proliferation of MCF-7 cells. Then we demonstrated that ODC-MPI-2 simultaneously perturbed the polyamine synthesis pathway and the cAMP pathway in MCF-7 cells. At last, we confirmed that ODC-MPI-2 has antitumor activity on breast cancer cells including a preference toward triple negative breast cancer cells. Altogether, our work demonstrated the potential of using ODC inhibitors and A_1_AR inhibitors as a combination therapy for breast cancer, and presented the potential value of the dual inhibitors of ODC and A_1_AR in treating breast cancer.

## Materials and Methods

### Cell Line and Cell Culture

The breast cancer cell lines MCF-7 and MDA-MB-231 were obtained from the China Center for Type Culture Collection (Wuhan, China). T-47D, MDA-MB-453, and BT-474 were purchased from Procell (Wuhan, China). The cells were bought right before the experiment was performed and authenticated by the providers with STR analysis. The MCF-7, MDA-MB-453, and MDA-MB-231 cells were cultured in Dulbecco’s modified Eagle’s medium (DMEM) containing 10% fetal bovine serum (FBS) (Cellmax, Beijing, China) and 1% antibiotic-antimycotic. The BT-474 cells were cultured in Roswell Park Memorial Institute (RPMI1640) medium supplemented with 20% FBS and 1% antibiotic-antimycotic. The T-47D cells were cultured in RPMI1640 medium supplemented with 10% FBS and 1% antibiotic-antimycotic. All cells were incubated at 37°C in a humidified incubator supplemented with 5% CO_2_.

### Chemicals

1, 3-dipropyl-8-cyclopentylxanthine (DPCPX), an A_1_R antagonist, was purchased from Sigma-Aldrich (USA). DPCPX was dissolved in dimethyl sulfoxide (DMSO). Eflornithine hydrochloride, hydrate (DFMO), an irreversible inhibitor of ODC, was purchased from MedChem Express (USA). DFMO was dissolved in ultrapure H_2_O. ODC-MPI-2 was purchased from MolPort (Latvia; MolPort ID: MolPort-002-603-288) and dissolved in DMSO.

### Protein Expression and Purification

The coding sequence of the full-length hODC1 was inserted in pET-28a and verified by DNA sequencing. The plasmid was transformed into the *Escherichia coli* strain BL21(DE3). The transformed strain was cultured in Luria Bertani (LB) broth medium with 50 µg/ml kanamycin at 37°C. When the OD_600_ value reached 0.4–0.6, protein expression was induced by 0.5 mM of IPTG (isopropyl *β*-D-1-thiogalactopyranoside) for 6 h at 28°C, 200 rpm. The bacteria were collected by centrifugation, resuspended in the lysis buffer (20 mM imidazole, 500 mM NaCl, 20 mM Na_2_HPO_4_, pH 7.6) and broken by ultra-sonication. The mixture was clarified by centrifugation at 12,000 rpm for 10 min at 4°C, and the supernatant was loaded to a HisTrap HP (GE Healthcare) column for the affinity capture of the His-tag hODC. The His-tag hODC was eluted with an imidazole gradient (50, 100, 150, 250, 350, and 500 mM) in the lysis buffer. All eluted fractions were analyzed by 15% SDS-PAGE, and the ones containing the target protein were subjected to a further purification with a Sephacryl S-200 HR column (GE Healthcare) in the storage buffer (10 mM HEPES, 20 mM NaCl, 0.1 mM EDTA, pH 7.6). The final product was collected and analyzed with 15% SDS-PAGE. The 6× His tag was not cleaved off in this study.

### Determination of ODC Activity With the CO_2_ Kit

To evaluate the release of CO_2_ from the decarboxylation of L-ornithine catalyzed by ODC, a carbon dioxide (CO_2_) assay kit (Biosino, Beijing, China) was used, and the assay was performed as previously described (19–21). Briefly, solution B (75 nM PLP with the indicated concentrations of ODC-MIP-2, with or without 50 μg ODC) was incubated at 37°C for 10 min in a 96-well plate before solution A (3 mM Ornithine) was added to solution B. Then the optical density (OD) was measured at 340 nm for 10 min on a Synergy™ H1 Microplate reader (BioTek Instruments, Inc., USA) at 25°C. Each sample had three replicates.

### Cell Growth Assay

Cells (3 × 10^3^ cells/well) were seeded in a 96-well plate and incubated for 12 h at 37°C for adherence. The cells were cultured in the medium supplemented with 10% fetal bovine serum (FBS) for 24–72 h after the addition of the indicated inhibitors. Then the medium was removed, and 100 µl of medium containing 10 μl of 3-(4,5-dimethylthiazol-2-yl)-2,5-diphenyltetrazolium bromide (MTT, Solarbio; 5 mg/ml) was added. The plate was incubated for 4 h at 37°C before 110 μl of dimethyl sulfoxide (DMSO) was added. After being incubated for 10 min at room temperature to dissolve the formazan crystals, the plate was placed in a Synergy™ H1 Microplate reader (BioTek Instruments, Inc., USA) to record the optical density (OD) of the samples at 490 nm. Each sample had three replicates. The viability percentage was calculated as: mean OD of the treated sample/mean OD of the control ×100. IC_50_ was defined as the concentration of the inhibitor at which cell growth was inhibited by 50% and calculated with an equation derived from a *log(inhibitor) vs. normalized response—Variable slope* method in GraphPad Prism 8.2.1. Although the depletion of higher polyamines (spermine and spermidine) needs long treatment time (≥72 h), we used 48 h in this assay and the other cellular assays (unless indicated otherwise) to focus on the combination efficacy instead of the polyamine depletion from one inhibitor.

### Cell Proliferation Assay

Cells (4 × 10^3^ cells/well) were seeded in a 96-well plate and incubated for 12 h at 37°C for adherence. Images of the same positions were taken for 48 h in an Incucyte S3 Live-Cell Analysis System (Essen BioScience, USA). The image data were processed with the IncuCyte ZOOM software and were expressed as the mean ± SD (n = 3) after normalization against the 0 h data.

### Wound Healing Assay

Cells (2.5 × 10^4^ cells/well) were seeded in a 96-well plate and incubated for 12 h at 37°C for adherence. A Wound Maker™ (Essen BioScience, USA) was used to scratch the confluent monolayers in a straight line when cells were 80–90% confluent. The floating cells were washed away with PBS for three times. Images of the same wound position were taken for 75 h in an Incucyte S3 Live-Cell Analysis System (Essen BioScience, USA). Relative wound density was calculated as the ratio of the cell density in the wound area against the cell density outside.

### Transwell Assay

2 × 10^4^ cells resuspended in 100 μl of serum-free medium with indicated inhibitors were cultured in the upper compartment of an 8.0 μm transwell chamber. Medium with 20% FBS was used as the chemoattractant in the lower chamber. After incubation for 24 h at 37°C, the cells that did not invade through the pores were removed with a cotton swab. The cells attached to the outside surface of the insert were stained in 1% crystal violet for 10 min before the insert was washed and photographed. To quantify the invaded cells, five independent visual fields were evaluated in NIH ImageJ for each sample.

### Determination of cAMP Levels

Cells (1.2 × 10^5^ cells/well) were seeded in a 24-well plate and incubated for 12 h at 37°C for adherence. Then the cells were treated for 6 h with the indicated inhibitors. The cells were lysed with RIPA lysis buffer (Sangon, China) and centrifuged at 4°C, 12,000 rpm for 10 min. cAMP levels were evaluated with the Direct Cyclic AMP Enzyme Immunoassay Kit (MSKBIO, China) as per the manufacturer’s instructions. Absorbance values were measured at 450 nm in a Synergy™ H1 Microplate reader (BioTek Instruments, Inc., USA).

### Quantitative Analysis of Polyamine Levels

Cells (8 × 10^5^ cells/well) were seeded in a 10 cm plate and incubated for 12 h at 37°C for adherence. After being treated by the indicated inhibitors for 72 h, the cells were washed with PBS and resuspended in 800 μl of lysis buffer (20 mM Tris-HCl, 150 mM NaCl, pH 8.0, 1% TritonX-100). The resuspended cells were kept on ice for 20 min and centrifuged at 4°C, 13,000 rpm for 10 min. The supernatants were collected, and the total protein concentration was determined by the BCA method (Sangon Biotech, China). Then 1.0 ml of sodium hydroxide (2 mol/L) and 5 μl of benzoyl chloride were added into the supernatant. The mixture was briefly vortexed and incubated for 20 min at 40°C before 1.0 ml of saturated sodium chloride solution was added to terminate the reaction. The sample was extracted with diethyl ether, and the ether phase was collected and dried *via* volatilization. The precipitate was dissolved in 1 ml of methanol, filtered, and immediately analyzed on an HPLC system (Thermo UltiMate3000) configured with a UV detector and a Symmetry C18 column (4.6 *250 mm, 5 μm). The HPLC condition was below: mobile phase: acetonitrile and water (40:60); column temperature: 30°C; flow rate: 1.0 ml/min; injection volume: 10 μl; detection wavelength: 238 nm.

### Drug Combination Analysis

MCF-7 cells were exposed to the combination of DFMO and DPCPX at the indicated concentrations. The interaction of drugs was calculated using the CompuSyn software (version 1.0, Combo-Syn In., US) based on the Chou-Talalay method (24). The calculated CI (combination index) values indicate the drug interactions. Briefly, CI > 1, CI = 1, CI < 1 represent antagonistic, additive, and synergic effects, respectively.

### Statistical Analyses

The cell assays were performed with multiple biological replicates (≥3). GraphPad Prism 8.2.1 was used for the statistical analyses. Data were shown as means ± standard deviation (SD). Significance analyses were carried out using Student’s *t*-test. The threshold for statistical significance was p < 0.05.

## Results

### The Combination of DFMO and DPCPX Has Synergistic Antitumor Activity

**Figure 1:**
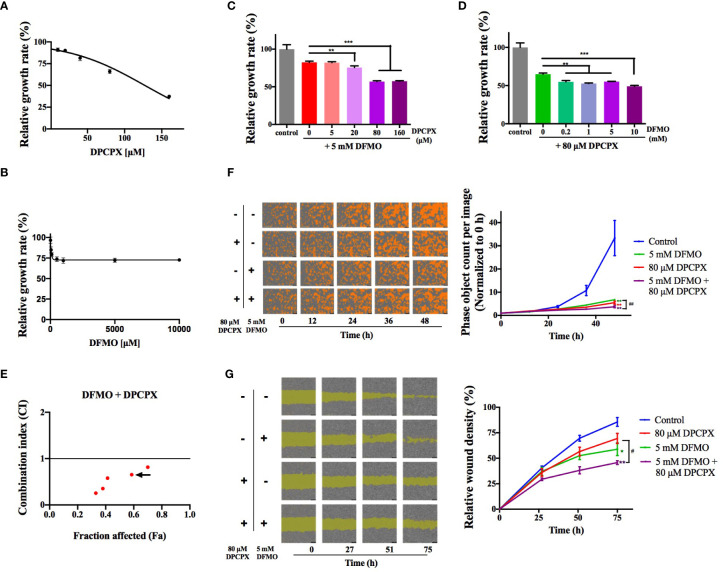
DFMO and DPCPX synergistically inhibit the growth and proliferation of MCF-7 cells. The relative growth data of MCF-7 cells from the MTT assay are shown for DPCPX **(A)** and DFMO **(B)**. Cells were seeded in 96-well plates and treated with 0–160 µM DPCPX or 0–10 mM DFMO for 48 h. Data are shown as mean ± SD (n = 3). **(C)** The relative growth of MCF-7 cells as determined by the MTT assay after the treatment with 5 mM DFMO and 0–160 µM DPCPX. Cells were seeded in 96-well plates and treated with the indicated inhibitors for 48 h. Data are shown as mean ± SD (n = 3). **p < 0.01; ***p < 0.001. **(D)** The relative growth of MCF-7 cells as measured by the MTT assay after treatment with 80 µM DPCPX and 0-10 mM DFMO. Cells were seeded in 96-well plates and treated with the indicated inhibitors for 48 h. Data are shown as mean ± SD (n = 3). **p < 0.01; ***p < 0.001. **(E)** The scatter plot of Combination Index (CI) *versus* fraction affected (Fa) based on the data from the MTT data. MCF-7 cells were treated with DFMO and DPCPX in the constant molar ratio 1:0.016. The CI and Fa values were calculated using the Chou-Talalay method with CompuSyn (24). CI > 1, CI = 1, CI < 1 represent antagonistic, additive, and synergic effects, respectively. The arrow marks the combination of 5 mM DFMO and 80 µM DPCPX (CI = 0.65). **(F)** Representative images from the cell proliferation assay performed on an IncuCyte S3 Live-Cell Analysis System (Essen BioScience, USA). Cells were seeded in 96-well plates and treated with the indicated dose combinations of DFMO and DPCPX. Cells are shown in orange. The quantified proliferation data with the IncuCyte ZOOM software are shown on the right as mean ± SD (n = 3). **p < 0.01 compared to the control group; ^##^ *p* < 0.01 compared to the DFMO group. **(G)** Representative images from the wound healing assay performed on an IncuCyte S3 Live-Cell Analysis System. Cells were seeded in 96-well plates and treated with indicated dose combinations of DFMO and DPCPX. The quantified relative wound densities from the IncuCyte ZOOM software are shown on the right as mean ± SD (n = 3). * and ** indicate *p* < 0.05 and *p* < 0.01 respectively compared to the control group. ^#^ *p* < 0.05 compared to the DPCPX group.

We asked whether simultaneously inhibiting ODC and A_1_AR has synergistic effects on inhibiting breast cancer cells. To that end, we tested the combined use of DFMO (an ODC-specific inhibitor) and DPCPX (a selective A_1_AR inhibitor) using the cell line MCF-7 (luminal A, ER+/PR+/HER2−). As shown in **Figures 1A, B**, MCF-7 cells were inhibited by DPCPX and DFMO in dose-dependent trends, but the single use of either one only showed moderate inhibition. The regular DFMO concentration used in previous cellular experiments was 2.5–20 mM (25). Therefore, we kept DFMO’s concentration at 5 mM to test the suitable DPCPX concentration for their combined use and noticed that 80 µM of DPCPX showed improved inhibition than their single use (**Figure 1C**). Our quantitative data from the Chou-Talalay method (24) further confirmed that the combination of 5 mM of DFMO and 80 µM DPCPX is a suitable choice for synergism (**Figures 1D, E** **)**. This synergistic inhibitory effect was further confirmed by the real-time cell growth assay (**Figure 1F**). Additionally, DFMO and DPCPX synergistically inhibited the migration of MCF-7 cells **(** **Figure 1G**). Taken together, these data confirmed that simultaneously inhibiting ODC and A_1_AR has synergistic effects on inhibiting the growth and proliferation of breast cancer cells *in vitro*.

### ODC-MPI-2 Is a Novel ODC Inhibitor

**Figure 2:**
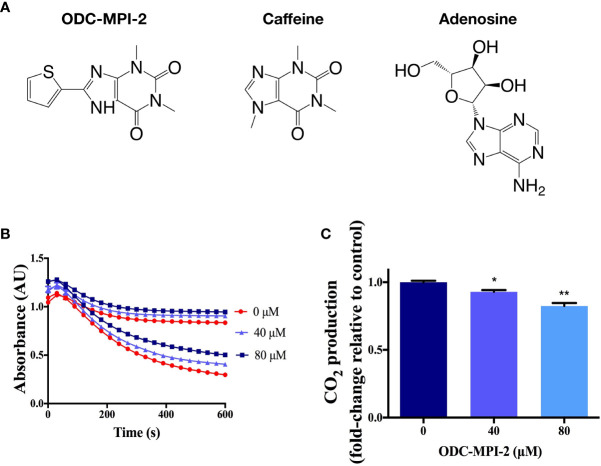
The confirmation of ODC-MPI-2 as an ODC inhibitor. **(A)** The structure of ODC-MPI-2, caffeine, and adenosine. **(B)** The release of CO_2_ from L-ornithine catalyzed by ODC was measured using the carbon dioxide assay (20, 21). The absorbance was measured at 340 nm. Data are shown as mean ± SD (n = 3). Each color represents the control sample (without ODC, higher values) and the test sample (with ODC, lower values), respectively. The relative CO_2_ productions at 600 s are shown in **(C)**. * and ** indicate *p* < 0.05 and *p* < 0.01 respectively compared to the control group.

In our recent work, we targeted the rate-limiting enzyme ODC (ornithine decarboxylase 1) in the polyamine metabolic network and established a computer-aided drug discovery protocol for screening novel ODC inhibitors (22). We discovered several novel multi-purpose ODC inhibitors that simultaneously inhibit ODC’s activity and ODC’s protein–protein interactions (22), and ODC-MPI-2 (**Figure 2A**) was one of them. Structurally, ODC-MPI-2 is a caffeine derivative and similar to adenosine (**Figure 2A**), which partially explains the activity of ODC-MPI-2 as an A_1_AR inhibitor, since caffeine was known to be an adenosine receptor antagonist (https://pubchem.ncbi.nlm.nih.gov/compound/caffeine). To further verify the inhibitory effect of ODC-MPI-2 on ODC’s enzymatic activity, we measured the release of CO_2_ by ODC using a carbon dioxide kit (20, 21). As shown in **Figures 2B, C** and **S1**, ODC-MPI-2 inhibited the production of CO_2_. Interfered by the solubility and the background absorption of the compound, the CO_2_ assay was not quantitative, but the result was consistent with our previous work, in which the inhibitory effect of ODC-MPI-2 was confirmed by HPLC (IC_50_ = 4.83 ± 1.17 µM) but under a different condition (22).

### ODC-MPI-2 Decreases Polyamine Level and Elevates cAMP Level in Cells

**Figure 3:**
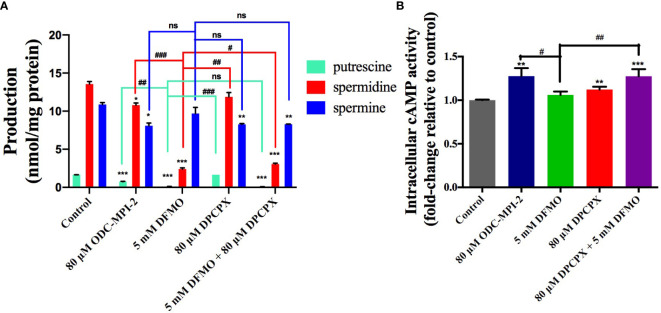
ODC-MPI-2 simultaneously perturbs the polyamine pathway and the cAMP pathway. **(A)** ODC-MPI-2 inhibited the synthesis of polyamines. Cells were treated with the indicated doses of different inhibitors for 72 h. The cells were lysed, and the polyamine amounts were quantified with an HPLC assay. The polyamine levels were expressed as the ratio between the polyamines and the amount of total proteins. The data are shown as mean ± SD (n = 3). *, ** and *** indicate *p* < 0.05, *p* < 0.01, and *p* < 0.001 respectively compared to the control group. ^#^, ^##^ and ^###^ indicate *p* < 0.05, *p* < 0.01, and *p* < 0.001 respectively compared to the DFMO group. **(B)** ODC-MPI-2 stimulated the production of cAMP. Cells were serum-starved and treated for 6 h with the indicated doses of different inhibitors. The cells were lysed, and the cAMP level was quantified using a Direct Cyclic AMP Enzyme Immunoassay Kit. Data are shown as mean ± SD (n = 3). ** and *** indicate *p* < 0.01 and *p* < 0.001 respectively compared to the control group. ^#^ and ^##^ indicate *p* < 0.05 and *p* < 0.01 respectively compared to the DFMO group. ns, not significant.

We noticed that ODC-MPI-2 was reported as an A_1_AR inhibitor previously (23), so we were interested in testing it as a dual inhibitor of ODC and A_1_AR to see if ODC-MPI-2 could simultaneously perturb polyamine synthesis and the downstream signaling pathway of A_1_AR. We quantified the polyamine content in MCF-7 cells after the treatment of different inhibitors (**Figures 3A** and **S2**). The data showed that DFMO significantly inhibited the synthesis of putrescine and spermidine, which was in agreement with previous reports (26). DPCPX slightly decreased the amount of spermine but did not cause significant changes on putrescine and spermidine. When combined, DFMO and DPCPX showed synergism in downregulating the cellular contents of putrescine, spermidine, and spermine. Similarly, ODC-MPI-2 decreased the cellular concentration of all polyamines.

A_1_AR preferentially couples to the G_i/o_ family of G-proteins, so the activation of A_1_AR activates the G_i/o_ protein, resulting in the inhibition of adenylate cyclase and the decrease in cAMP level (27). Correspondingly, inhibiting A_1_AR would induce the elevation of cellular cAMP level. Indeed, we noticed DPCPX caused the elevation of cAMP in MCF-7 cells, and the combination of DFMO and DPCPX caused even more significant elevation (**Figures 3B** and **S3**), indicating a synergistic effect of these two inhibitors. In agreement with this synergistic result, ODC-MPI-2 also stimulated the elevation of cellular cAMP level (**Figure 3B**).

Taken together, these data indicated that the combination of DFMO and DPCPX has synergistic effects on perturbing both the polyamine synthesis pathway and the cAMP pathway. And as a dual inhibitor of ODC and A_1_AR, ODC-MPI-2 simultaneously perturbs the polyamine metabolism network and the cAMP pathway as well.

### 3.4 ODC-MPI-2 Has Good Antitumor Potency on Breast Cancer Cells

**Figure 4:**
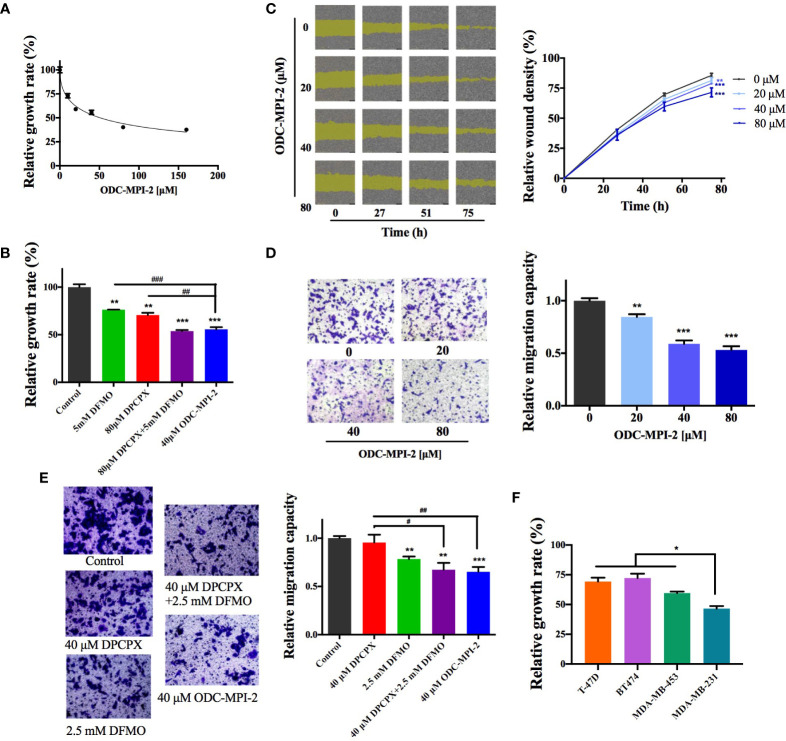
ODC-MPI-2 has good antitumor potency on breast cancer cell lines. **(A)** Relative growth of MCF-7 cells as measured by the MTT assay after treatment of ODC-MPI-2. Cells were seeded in 96-well plates and treated with 0-160 μM ODC-MPI-2 for 48 h. Data are shown as mean ± SD (n = 3). **(B)** Relative growth of MCF-7 cells as measured by the MTT assay after the treatment of different inhibitors. Cells were seeded in 96-well plates and treated with 5 mM DFMO, 80 μM DPCPX, or 40 μM ODC-MPI-2 for 48 h. Data are shown as mean ± SD (n = 3). ** and *** indicate *p* < 0.01 and *p* < 0.001 respectively compared to the control group. ^##^ and ^###^ indicate *p* < 0.05 and *p* < 0.01 respectively compared to the ODC-MPI-2 group. **(C)** Representative images from the wound healing assay obtained from an IncuCyte S3 Live-Cell Analysis System (Essen BioScience, USA). Cells were seeded in 96-well plates and treated with 0–80 μM ODC-MPI-2. The relative wound densities were quantified with the IncuCyte ZOOM software and shown on the right as mean ± SD (n = 3). ** and *** indicate *p* < 0.01 and *p* < 0.001 respectively compared to the 0 μM group, which is a same control used in **Figure 2G**. **(D)** Cell migration studies of MCF-7 cells treated with ODC-MPI-2 from the Transwell assay. The cells were treated by 0-80 μM ODC-MPI-2 for 24 h. The representative images are shown on the left and the quantified data on the right. Data are shown as mean ± SD (n = 5). ** and *** indicate *p* < 0.01 and *p* < 0.001 respectively compared to the 0 μM group. **(E)** The cell migration capacity of MCF-7 cells treated with DFMO, DPCPX, and ODC-MPI-2 from the Transwell assay. The representative images are shown on the left and the quantified data on the right. Data are shown as mean ± SD (n = 5). **and ** indicate *p* < 0.05 and *p* < 0.01 respectively compared to the control group. ^##^ and ^###^ indicate *p* < 0.01 and *p* < 0.001 respectively compared to the 40 μM ODC-MPI-2 group. **(F)** Relative growth of different breast cancer cells as measured by the MTT assay after treatment with ODC-MPI-2. Cells were seeded in 96-well plates and treated with 40 μM ODC-MPI-2 for 48 h. The Data are shown as mean ± SD (n = 3). *p < 0.05 compared to the MDA-MB-231 group.

We continued to investigate if ODC-MPI-2 inhibits the growth and proliferation of breast cancer cells. In the MTT assay (**Figure 4A**), ODC-MPI-2 efficiently inhibited the growth and proliferation of MCF-7 cells at micromolar concentrations (fitted EC_50_ = 50.9 µM). Furthermore, at comparable concentrations, ODC-MPI-2 showed better inhibitory effects than DFMO or DPCPX in the MTT assay (**Figure 4B**). The wound healing assay (**Figure 4C**) and the transwell assay (**Figure 4D**) further showed that ODC-MPI-2 effectively inhibited the proliferation and migration of MCF-7 cells. Similarly, at comparable concentrations, ODC-MPI-2 showed better inhibitory effects than DFMO and DPCPX in the transwell assay (**Figure 4E**). Lastly, we tested the inhibitory potency of ODC-MPI-2 on different types of breast cancer cells. As shown in **Figure 4F**, the data showed that ODC-MPI-2 inhibited different breast cancer cells with different potencies. Interestingly, ODC-MPI-2 showed obvious preference in inhibiting the triple-negative cell line MDA-MB-231 (ER−/PR−/HER2−), although it also inhibited the other cell lines (BT474, ER+/PR+/HER2+; T-47D, ER+/PR+/HER2−; MDA-MB-453, ER−/PR−/HER2+).

## Discussion

A_1_AR has important cellular functions by incurring down-stream signaling pathways upon the binding of adenosine. The inhibition of A_1_AR activates the adenylate cyclase, inducing the elevation of cellular cAMP level (27). As a ubiquitous second messenger, cAMP plays important roles in regulating cell proliferation and differentiation. In recent years, cAMP elevating agents were proven to have excellent antitumor activity by inducing cell apoptosis and/or cell cycle arrest of tumor cells (28). Additionally, cAMP elevating agents showed enhanced anti-tumor activity when used in combination with other anti-tumor agents. For example, the activation of the cAMP pathway enhanced the apoptosis of cancer cells in oncolytic virotherapy (29), and Leptin enhanced the anti-proliferative effect of cAMP elevating agents (30). Specifically, the chemopreventative agent resveratrol was proved to be an agonist for the cAMP pathway in breast cancer cells (31).

Polyamines are endogenous cationic ligands involved in many physiological processes including regulating chromatin structure, gene expression, transcription, and protein function (12, 32). In breast cancer, the polyamine metabolic network is deregulated mainly *via* the up-regulation of the polyamine synthetic enzyme ODC (16). Although polyamine decreasing agents are promising in treating cancers (20, 21), their combinational use with other drugs is emerging as a more attractive therapeutic strategy (18). A recent review of the clinical trials between 1989 and 2007 revealed that although DFMO was active against recurrent gliomas as a single agent, its combination with procarbazine, lomustine, vincristine (PCV) showed improved efficacy (14). In neuroblastoma mice, the combination of DFMO and AMXT 1501, a polyamine uptake inhibitor, was more beneficial than DFMO monotherapy (33). Currently, the combination therapy strategy is under intensive investigations against various tumors by combining DFMO with other agents such as AMXT 1501 (NCT03536728), bortezomib (NCT02139397), etoposide (NCT01059071), lomustine (NCT02796261), and dinutuximab/irinotecan/temozolomide (NCT03794349).

In this work, we demonstrated that the combined use of the ODC inhibitor DFMO and the A_1_AR inhibitor DPCPX has synergistic anti-tumor activity in breast cancer cells. To our knowledge, this is the first time that these two signaling pathways are shown to have synergistic effects in inhibiting the growth and proliferation of tumor cells. Furthermore, we demonstrated that ODC-MPI-2, as an A_1_AR inhibitor and a novel multi-purpose ODC inhibitor, has good anti-tumor activity against breast cancer cells. Our result resonates with a previous report showing that spermine increased adenosine’s binding affinity to A_1_AR (34), indicating the synergistic effect of the polyamine metabolism network and the A_1_AR signaling pathway. A major surprise is that our data showed that ODC-MPI-2 had good preference for inhibiting the triple-negative breast cancer (TNBC) cells (MDA-MB-231) over the other types (**Figure 4F**). Considering that the cure of TNBC is a major challenge in the treatment of breast cancer, our finding could provide an exciting strategy. Recently, Geck et al. noticed that DFMO treatment sensitized TNBC cells to chemotherapy because ODC levels were elevated in TNBC patient samples (17), and this reason could be also true in our work. An additional possibility is that the A_1_AR signaling pathway in TNBC is also up-regulated. It will be interesting to reveal the exact mechanism in the future.

There are several caveats in our current work. Our data showed that the inhibition of A_1_AR perturbed the synthesis of spermine, and the simultaneous inhibition of ODC and A_1_AR leads to significant decrease of total polyamines (putrescine, spermidine, and spermine) which in turn inhibits cellular growth. But the detailed mechanism of this finding is open for further investigations. Another caveat is that the synergism of DFMO and DPCPX was only moderate under the tested condition. It is possible that the synergism would be more obvious at different inhibitor combinations, under different experimental conditions, and on different cell lines. However, this caveat might highlight the value of dual inhibitors since ODC-MPI-2, at a lower concentration than either one of them, was as effective as the combination of these two specific inhibitors. Considering that ODC-MPI-2 is a moderate inhibitor of A_1_AR and ODC, and its specificity is not verified at cellular level, we suppose that optimized dual inhibitors could have more significant efficacy. Lastly, the preference of ODC-MPI-2 toward inhibiting TNBC cells needs to be further verified with more cell lines and in *in vivo* models.

## Conclusions

This work demonstrated the potential value of simultaneously inhibiting polyamine synthesis and adenosine receptors in treating tumor cells. Since adenosine receptors and polyamine metabolism are both closely related in other conditions like DNA methylation and epigenetics, their dual inhibitors might have wider applications. For instance, it is suggested that cAMP elevating agents are useful in treating Alzheimer’s disease (28). We (32) and others (35) showed that elevated polyamine levels promote protein aggregation, which contributes to the incidence of Alzheimer’s disease. As such dual inhibitors of polyamine synthesis and A_1_AR could be good candidates for treating Alzheimer’s disease. In summary, our findings here may have broad applications in the future.

## Data Availability Statement

The original contributions presented in the study are included in the article/**Supplementary Material**. Further inquiries can be directed to the corresponding author.

## Author Contributions

SL conceived the study, participated in methodology design, supervised the project, and draft the manuscript. HM carried out the molecular and cellular experiments, analyzed the results, and participated in drafting the manuscript. QL participated in the analysis of the data and drafting the manuscript. JW participated in the molecular experiments. JP participated in the cellular experiments. ZS participated as the acquirer of the experimental resources and the coordination of the project. All authors contributed to the article and approved the submitted version.

## Funding

SL was supported by the grants from National Natural Science Foundation of China (31971150, 31670768), Department of Science and Technology, Hubei Provincial People’s Government (2019CFA069), and Wuhan Municipal Science and Technology Bureau of China (2018060401011319).

## Conflict of Interest

The authors declare that the research was conducted in the absence of any commercial or financial relationships that could be construed as a potential conflict of interest.

## Abbreviations

A1AR, A1 adenosine receptor; ODC, ornithine decarboxylase 1; DFMO, *α*-difluoromethylornithine; GPCR, G protein-coupled receptors; AR, adenosine receptor; DMEM, Dulbecco’s modified Eagle’s medium; FBS, fetal bovine serum; DPCPX, 1, 3-dipropyl-8-cyclopentylxanthine; CI, combination index; TNBC, triple-negative breast cancer.
