# Supplementary material for: Dual Inhibition of Ornithine Decarboxylase and A1 Adenosine Receptor Efficiently Suppresses Breast Tumor Cells
Source: Front Oncol. 2021 Mar 11;11:636373. doi: 10.3389/fonc.2021.636373 (PMC7991832; doi:10.3389/fonc.2021.636373)
Supplement: Supplementary file 1 [file DataSheet_1.pdf]

Supplementary Data

Dual inhibition of ornithine decarboxylase and A_1 adenosine receptor efficiently suppresses breast tumor cells

Hongyan Ma[1,2,3], Qizhang Li[1,2], Jing Wang[1,2,3], Jing Pan[1,2,3], Zhengding Su[1,2], Sen Liu[1,2,3,*]

[1] National "111" Center for Cellular Regulation and Molecular Pharmaceutics, Key Laboratory of Industrial Fermentation (Ministry of Education), Hubei University of Technology, Wuhan 430068, China

[2] Institute of Biomedical and Pharmaceutical Sciences, Hubei Key Laboratory of Industrial Microbiology, Hubei University of Technology, Wuhan, 430068, China

[3] Hubei Key Laboratory of Tumor Microenvironment and Immunotherapy, Medical College of China Three Gorges University, Yichang 443002, China

[*]Corresponding author: senliu.ctgu@gmail.com, ORCID: 0000-0001-5182-7241.

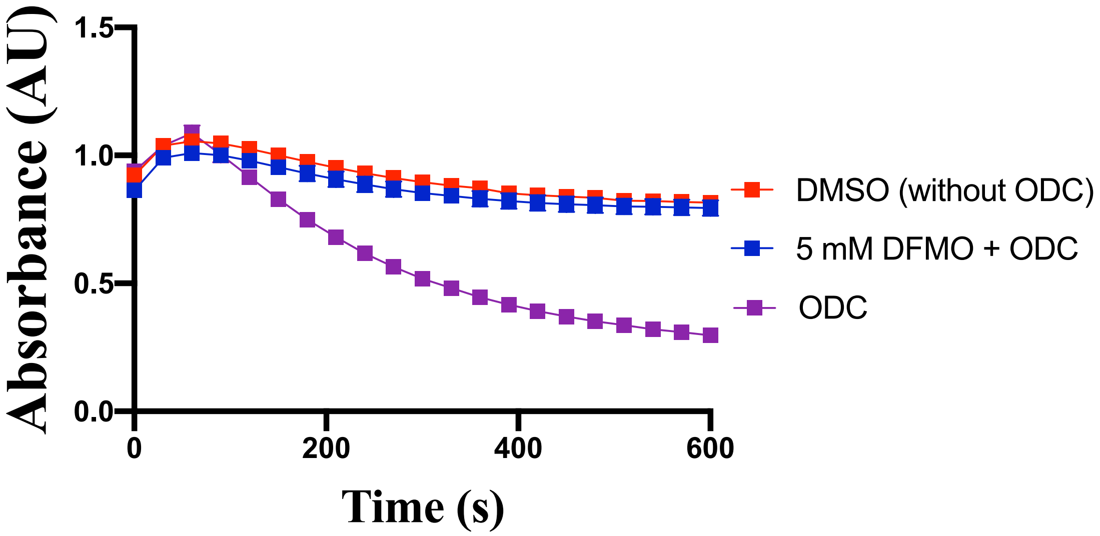

Figure S1. The inhibition of DFMO on ODC activity measured by the CO_2 kit.

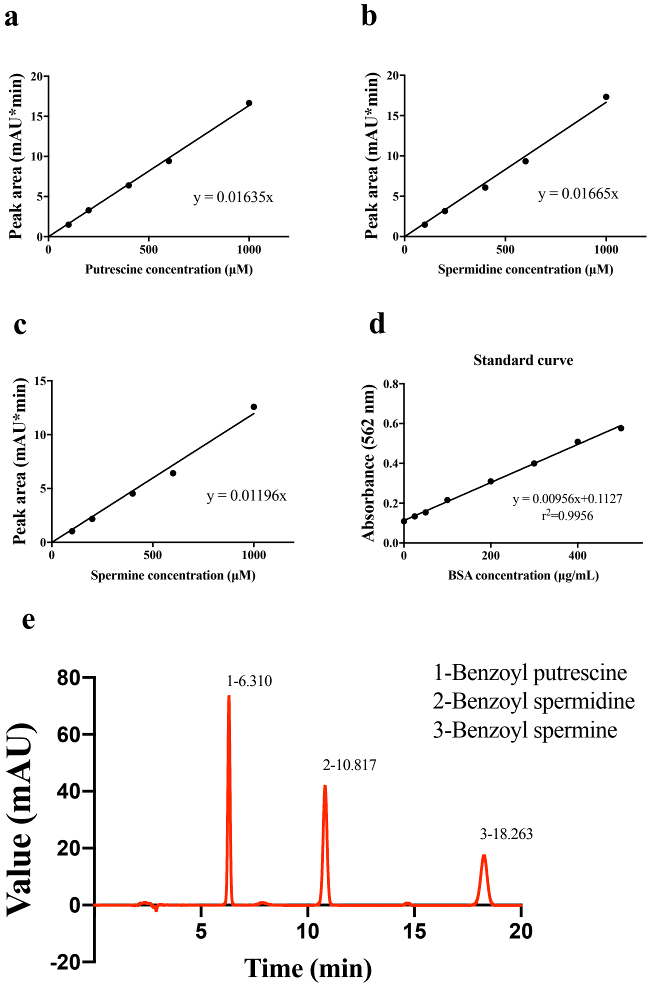

Figure S2. The standard curves for the HPLC quantification of polyamines (**a**, **b**, **c**) and total proteins (**d**). An HPLC spectrum of the mixed polyamine samples is shown in **e**.

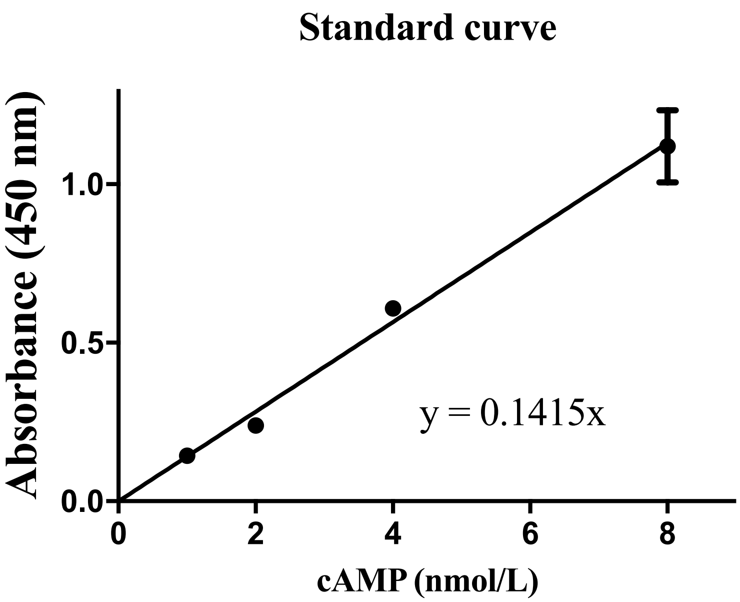

Figure S3. The standard curve for cAMP quantification.
